# Modeling the neuropsychiatric manifestations of Lowe syndrome using induced pluripotent stem cells: defective F-actin polymerization and WAVE-1 expression in neuronal cells

**DOI:** 10.1186/s13229-018-0227-3

**Published:** 2018-08-15

**Authors:** Jesse Barnes, Franklin Salas, Ryan Mokhtari, Hedwig Dolstra, Erika Pedrosa, Herbert M. Lachman

**Affiliations:** 1 Department of Genetics, Albert Einstein College of Medicine, Bronx, New York, USA; 2 Department of Psychiatry and Behavioral Sciences, Albert Einstein College of Medicine, Bronx, New York, USA; 3 Department of Neuroscience and Physiology, SUNY Upstate Medical University, Syracuse, New York, USA; 4 Swammerdam Institute of Life Sciences, University of Amsterdam, Amsterdam, Netherlands; 5 Department of Neuroscience, Albert Einstein College of Medicine, Bronx, New York, USA; 6 Department of Medicine, Albert Einstein College of Medicine, Bronx, New York, USA

**Keywords:** Lowe syndrome, Dent disease, OCRL, INPP5B, Induced pluripotent stem cells, Autism, Developmental, Intellectual, Renal, Cataract

## Abstract

**Background:**

Lowe syndrome (LS) is a rare genetic disorder caused by loss of function mutations in the X-linked gene, *OCRL*, which codes for inositol polyphosphate 5-phosphatase. LS is characterized by the triad of congenital cataracts, neurodevelopmental impairment (primarily intellectual and developmental disabilities [IDD]), and renal proximal tubular dysfunction. Studies carried out over the years have shown that hypomorphic mutations in *OCRL* adversely affect endosome recycling and actin polymerization in kidney cells and patient-derived fibroblasts. The renal problem has been traced to an impaired recycling of megalin, a multi-ligand receptor that plays a key role in the reuptake of lipoproteins, amino acids, vitamin-binding proteins, and hormones. However, the neurodevelopmental aspects of the disorder have been difficult to study because the mouse knockout (KO) model does not display LS-related phenotypes. Fortunately, the discovery of induced pluripotent stem (iPS) cells has provided an opportunity to grow patient-specific neurons, which can be used to model neurodevelopmental disorders in vitro, as demonstrated in the many studies that have been published in the past few years in autism spectrum disorders (ASD), schizophrenia (SZ), bipolar disorder (BD), and IDD.

**Methods:**

We now report the first findings in neurons and neural progenitor cells (NPCs) generated from iPS cells derived from patients with LS and their typically developing male siblings, as well as an isogenic line in which the *OCRL* gene has been incapacitated by a null mutation generated using CRISPR-Cas9 gene editing.

**Results:**

We show that neuronal cells derived from patient-specific iPS cells containing hypomorphic variants are deficient in their capacity to produce F-filamentous actin (F-actin) fibers. Abnormalities were also found in the expression of WAVE-1, a component of the WAVE regulatory complex (WRC) that regulates actin polymerization. Curiously, neuronal cells carrying the engineered OCRL null mutation, in which OCRL protein is not expressed, did not show similar defects in F-actin and WAVE-1 expression. This is similar to the apparent lack of a phenotype in the mouse *Ocrl* KO model, and suggests that in the complete absence of OCRL protein, as opposed to producing a dysfunctional protein, as seen with the hypomorphic variants, there is partial compensation for the F-actin/WAVE-1 regulating function of OCRL.

**Conclusions:**

Alterations in F-actin polymerization and WRC have been found in a number of genetic subgroups of IDD and ASD. Thus, LS, a very rare genetic condition, is linked to a more expansive family of genes responsible for neurodevelopmental disorders that have shared pathogenic features.

**Electronic supplementary material:**

The online version of this article (10.1186/s13229-018-0227-3) contains supplementary material, which is available to authorized users.

## Background

Lowe syndrome (LS) (OMIM #300535) is a rare genetic disorder (~ 1/500,000 males) caused by loss of function mutations in the X-linked gene, *OCRL* (*OCRL-1; INPP5F*) [1–4]. It is characterized by the triad of congenital cataracts, neurodevelopmental impairment, and renal proximal tubular dysfunction [5]. Hypotonia, short stature, epilepsy, and behavioral problems (tantrums and stereotypy [complex repetitive behaviors]) are commonly found as well.

*OCRL* codes for a 901 amino acid protein, inositol polyphosphate 5-phosphatase, which is a key factor in endosome recycling and actin polymerization [6, 7]. A more moderate form of OCRL deficiency known as Dent-2 disease is dominated by the renal manifestations [8]. OCRL catalyzes the removal of the 5′ phosphate from phosphatidylinositol 4,5-bisphosphate (PI(4,5)P2), phosphatidylinositol 1,4,5-trisphosphate, and inositol 1,3,4,5-tetrakisphosphate [9–11].

There is extensive *OCRL* allelic heterogeneity in LS, which is primarily caused by hypomorphic missense mutations that lead to markedly reduced 5-phosphatase activity [12–14]. More than 90% of mutations occur in exons 9–14, and 18–24, which code for the phosphatase and ASH-RhoGAP domains, respectively [14, 15]. Hypomorphic variants in the ASH-RhoGAP domain affect the recruitment of OCRL to early endosomes by impaired binding to APPL1 and RAB5 [7]. Approximately 6% of LS cases are caused by deletions affecting the phosphatase domain [16, 17]. Complete *OCRL* deletions of the gene are rare. Paradoxically, one such deletion resulted in intellectual disability, but no renal disease [16]. On the other hand, a complete deletion in two other cases resulted in LS [18, 19]. In addition, Hichri et al. found a Dent disease patient with a frameshift deletion in exons 3 and 4 who did not have intellectual disability or congenital cataracts [14]. These findings suggest that genetic background and/or compensation by OCRL paralogs, such as *INPP5B*, could affect the clinical presentation of individuals with *OCRL* deficiency. Compensation by *Inpp5b* has been suggested as a possible mechanism for the absence of LS-related clinical phenotypes in the mouse *Ocrl* knockout (KO) model [20].

The molecular basis of LS has been primarily investigated in fibroblasts and immortalized cell lines (e.g., HeLa; Cos-7 cells). OCRL deficiency impairs the recycling of various receptors by reducing the trafficking of early endosomes to late endosomes [6, 7]. OCRL interacts with clathrin, and null and hypomorphic mutations lead to impaired clathrin-mediated endocytosis [2, 21, 22]. This can impair the recycling of various receptors, including megalin, which is responsible for the low molecular weight proteinuria and aminoaciduria seen in LS patients [6, 22].

So far, the effects of *OCRL* loss of function mutations on neuronal function and the brain have not been adequately investigated, but a few interesting clinical and preclinical findings are emerging. In LS children, delayed myelination, dilated perivascular spaces, and the development of multiple small cystic lesions in deep, periventricular white matter have been observed [23]. In zebrafish, *ocrl1* deficiency increases the susceptibility to heat-induced seizures, and causes cystic brain lesions and reduced Akt signaling. In addition, there is an increase in apoptosis and reduced proliferation in neural tissue. These effects appear to be due to deficits in clathrin-mediated membrane trafficking [24]. Mouse models have been developed, but, as noted above, *Ocrl* null mice are asymptomatic [20, 25].

Although these studies have been instrumental in helping to elucidate the molecular effects of OCRL deficiency and its pathological and clinical consequences in non-neuronal cells, there is a gap in understanding the effects of OCRL deficiency at a cellular level in neurons and, perhaps, other cell types found in the brain. In addition, because of the unique structure of neurons, loss of function *OCRL* mutations could lead to abnormalities and features not seen in non-neuronal cell types, such as neurite outgrowth, axonal transport, and dendritic spine morphology. This can now be addressed using induced pluripotent stem (iPS) cell technology, which is transforming the study of neurodevelopmental and neuropsychiatric disorders through the capacity to produce patient-specific neurons, astrocytes, oligodendrocytes, and microglia [26–40]. In addition, disease modeling using iPS cells can also be accomplished by altering the expression of relevant genes through KO or RNA interference (RNAi), as we and others have done for the autism spectrum disorders (ASD) and schizophrenia (SZ) candidate gene *CHD8* [32, 41, 42].

We now report preliminary findings on NPCs and neurons derived from iPS cells made from boys with LS and their typically developing siblings, as well as an *OCRL* KO iPS cell line generated from a control line using CRISPR-Cas9 gene editing. A defect in filamentous actin (F-actin) and WAVE-1 formation was found in neuronal cells derived from the patient samples.

Interestingly, in a finding similar to the absence of an observable phenotype in the mouse *Ocrl* KO model, a defect in WAVE-1 expression was not observed in the *OCRL* engineered KO line. This could be due to differences in the expression of OCRL-deficient phenotypes between hypomorphic variants, in which a mutated protein is produced, compared with the complete absence of OCRL protein, the latter of which allows for rescue by a compensatory pathway.

## Methods

### Subjects

The study and consent forms were approved by the Albert Einstein College of Medicine (AECOM). A diagnosis of LS was made during infancy in each patient based on clinical findings, fibroblast OCRL enzyme activity, and ultimately by genotyping.

### Development of iPS cells from peripheral blood CD34+ cells

iPS cell lines were generated from human peripheral blood CD34+ cells with a CytoTune-iPS 2.0 Sendai Reprogramming Kit (Invitrogen) following the manufacturer’s protocol. Briefly, frozen PBMCs were thawed 2 days before reprogramming (day − 2) and cultured in STIF medium [43]. On day 0, CD34+ cells were flow-sorted by FACSAria II (bipolar disorder; BD) and transduced with Sendai virus vectors containing Klf4–Oct3/4–Sox2, cMyc, and Klf4 in the presence of 4 μg/mL of polybrene. Three days after transduction, the transduced cells were plated on a Matrigel-coated 24-well plate in StemSpan SFEM medium (STEMCELL Technologies). On day 7, half of the StemSpan SFEM medium was replaced, and on day 8, the culture medium was completely replaced. Thereafter, culture medium was changed every 2 days from days 2–7, then changed daily from day 8. The iPS cell-like clones were picked and passaged by mechanical dissection from day 21 to day 28. FACS analysis of pluripotent markers (SSEA3, SSEA4, TRA-1-60, TRA-1-81), in vitro differentiation and immunohistochemical detection of three germ-layer markers (α-fetoprotein, α-smooth muscle actin and β-III tubulin), RT-PCR assay for virus gene integration, and karyotyping were performed on each iPS cell clone to ensure that integration-free iPS cells with the capacity to differentiate into all three germ layers were generated.

### Generating neural progenitor cells (NPCs) and neurons from iPS cells

The NPC protocol was adapted from the STEMCELL Technologies, STEMdiff™ Neural Induction Protocol with slight modifications. Briefly, iPS cells were maintained in mTeSR1 with daily feeding until near confluence. At the start of the experiment, differentiated cells were manually removed and the remaining un-differentiated cells were washed with PBS. The cells were incubated with gentle dissociation reagent (Stemcell Tech) for 8–10 min at 37 °C. Cells were dislodged by pipetting with a sterile 1-ml pipet tip (using a P1000 pipet) and collected in a 15-ml tube. The cell culture plate was rinsed with DMEM/F12 and added to the tube containing the cell suspension. Viable cells were treated with Trypan Blue and counted manually using a hemocytometer. Cells were then centrifuged at 300×*g* for 5 min. Supernatant was carefully aspirated, and cell pellet was re-suspended in STEMdiff™ Neural Induction Medium + 10 uM Y-27632 (ROCK inhibitor) Medium + 10 uM Y-27632 (ROCK inhibitor) to obtain a final concentration of 10^6^ cells/ml. Two milliliters of cell suspension were aliquoted into one well of a six-well plate, pre-coated with matrigel. Cells were fed daily for 6–7 days in STEMdiff™ Neural Induction medium without Y-27632. NPCs are ready for passage when cultures are approximately 90% confluent. NPCs are washed with DMEM/F12 and 1 ml of accutase was added to each well for 7 min at 37 °C. Cells were dislodged with a sterile 1-ml pipet tip and collected in a 15 ml tube. The cell culture plate was rinsed with DMEM/F12, and remaining NPCs were transferred to the bulk cell suspension. Viable cells were stained with Trypan Blue and counted using a hemocytometer. Cells were then centrifuged at 300×*g* for 5 min. Supernatant was carefully aspirated, and cell pellet was re-suspended in STEMdiff™ Neural Induction Medium + 10 uM Y-27632. Cells were plated at a density of 1.5 × 10^6^ cells/well in a six-well plate, pre-coated with PORN/Laminin. They were subsequently fed daily with STEMdiff™ Neural Induction Medium without Y-27632. NPCs were induced to differentiate into neurons at passage four.

Once NPCs reached ~ 50% confluence, neural differentiation was initiated by withdrawing FGF2 and adding NBF media supplemented with fresh growth factors as follows: WNT3A (100 ng/ml) (R&D Systems), BDNF (10 ng/ml), GDNF (10 ng/ml), IGF-1 (10 ng/ml) (PeproTech), and cAMP 1 μM (Sigma), as previously described [32], which produces a heterogeneous mix of glutamatergic neurons and GABAergic neurons.

### CRISPR-Cas9 gene editing

The *OCRL* KO line (690KO) was generated from a previously generated control (690C) unrelated to the LS subjects using CRISPR-Cas9 gene editing, as described by Ran et al. [44]. Guide sequences coding for a region on exon 6 were cloned into the Bbs1 site of pSpCas9n(BB)-2A-Puro (Addgene catalog #48139) using the guide RNA encoding sequences CACCGTACCAGAAATTAGACACTA (top strand) and AAACTAGTGTCTAATTTCTGGTAC (bottom strand), which were annealed prior to ligation into the linearized plasmid. Human iPS cells from a typically developing control were cultured and fed daily in mTeSR1 (Stem Cell technologies) on Matrigel (BD) coated plates at 37 °C/5% CO_2_/85% in a humidified incubator. Cells were maintained in log phase growth, and differentiated cells were manually removed before starting the experiment. iPS cells were exposed to 10 uM ROCK Inhibitor for ~ 4 h to improve cell survival during nucleofection. After 4 h, growth medium was aspirated and the cells were rinsed with DMEM/F12. iPS cells were dissociated into single cells with accutase and harvested by centrifugation. Nucleofection was performed using the Amaxa-4D Nucleofector Basic Protocol for Human Stem Cells (Lonza) according to the manufacturer’s instructions. Briefly, 8 × 10^5^ cells and 5 μg of plasmid were nucleofected using the P3 Primary Cell 4D-Nucleofector X Kit L with program CA-137. Cells were re-suspended in mTeSR1 + 10 uM ROCK Inhibitor and placed in one well of a six-well Matrigel-coated plate. The following day, cells were fed with fresh mTeSR1 and were subsequently fed with fresh medium daily. On days 4–14, cells were exposed to 0.5 μg/ml puromycin for 6 h. Puromycin-resistant colonies were picked and expanded in mTeSR1 without further puromycin treatment. DNA was analyzed to identify clones with frame shift mutations (see below).

### DNA and cDNA sequencing

Total cellular DNA was isolated using Gentra Puregene Blood Kit (Qiagen catalog# 158445). Total RNA was extracted using miRNeasy Mini Kit (Qiagen, catalog# 217004) according to the manufacturer’s instructions (Qiagen). An additional treatment with DNase1 (Qiagen, Valencia, CA) was included to remove genomic DNA. cDNA for sequencing was generated from RNA by RT-PCR (OneStep RT-PCR Kit, Qiagen, catalog# 210210 Valencia, CA) (see primers below). Genomic DNA was amplified using Taq DNA Polymerase (Invitrogen, catalog# 18038–042) using primers that flank the patient-specific and KO alleles (OCRL_LS100/300 for LS100 and LS300; OCRL_LS500 for LS500; OCRL_KO for the CRISPR-engineered line; see below for primer sequences). For the splice junction analysis, cDNA was amplified using exon 22 and exon 24 primers (LS300cDNA), and exon 23 and 24 primers (LS100cDNA). Amplicons were purified using a QIAquick PCR Purification Kit (Qiagen, catalog# 28104) according to the manufacturer’s instructions. DNA and cDNA were sequenced by the standard Sanger dideoxy chain termination method using nested primers (LS100seq, LS300seq), or one of the PCR primers.

### Western blotting

Proteins were prepared with ProteoExtract Complete Mammalian Proteome Extraction Kit (Millipore cat# 539779) according to the manufacturer’s protocol. Protein concentrations were verified using the Qubit™ Protein Assay kit (Invitrogen cat#Q33211). Briefly, 20–100 μg of protein were denatured with the addition of Laemmli buffer and 2-mercaptoethanol and boiled for 5 min. Samples were loaded onto a 12% precast polyacrylamide gel (BIO-RAD cat#456-1044). Gel electrophoresis was set at constant voltage (80 V) for the first 30 min and 200 V for the remainder of the run. The running buffer was in 1X TrisGlycine/SDS buffer. After separation by electrophoresis, proteins were bound to a nitrocellulose membrane (BIO-RAD cat# 162-0146). Electrophoretic transfer was set at constant voltage (70 V) for 2 h at 4 °C in 1X TrisGlycine buffer containing 20% methanol. After transfer, membranes were blocked in 5% milk with gentle agitation for 1 h at room temperature. Membranes were then incubated overnight with gentle agitation at 4 °C with primary antibody of interest. Following primary antibody incubation, membranes were washed three times with gentle agitation in 1X TBS/T buffer (20 mM Tris Base, 0.136 M NaCl, 0.1% Tween-20). Membranes were then incubated with secondary antibody plus anti-biotin for 1 h at room temperature with gentle agitation. Membranes were washed again, as above, and subsequently incubated with SuperSignal™ West Dura Extended Duration Substrate (Thermo Scientific cat# 34075) for 5 min at room temperature with gentle agitation. Immediately thereafter, membranes were exposed to blue autoradiograph film for analysis.

### Immunocytochemistry (ICC)

Cells were fixed in 10% buffered formalin phosphate for 10 min at 4 °C, then permeabilized at room temperature for 15 min in 1% Triton X-100 in PBS. Samples were blocked in 1% BSA (10 mg/ml), 5% donkey serum, and 0.1% Triton X-100 for 45 min at room temperature. Samples were then incubated for 1 h at room temperature with primary antibodies. Samples were washed seven times with Rinse Solution (5% donkey serum, 1% BSA; 5 min per wash). Secondary antibodies (Alexa Fluor 488 anti-rabbit, Alexa Fluor 568 anti-mouse, 1:300, Thermo Scientific) were applied for 45 min at room temperature in the dark (excluding F-actin, since conjugated phalloidin:FITC requires no secondary). Each sample was then washed five times with Rinse Solution for 5 min each. Coverslips were applied with ProLong Gold™ antifade reagent with DAPI (cat# P36931, Invitrogen) and visualized after 24 h.

Neurons were grown on 12-mm coverslips, and NPCs were grown on four-well chamber slides pre-coated with PORN/Laminin. All images were taken with AxioVert 200 M inverted fluorescence microscope equipped with a Plan-Apochromat × 63/1.40 Oil DIC Objective and HBO 100 microscope illumination system. Alexa-Fluor 488 fluorescence was measured with a GFP-470-nm excitation filter/509-nm emission filter. Alexa-Fluor 568 fluorescence was measured with a Rhodamine-540-nm excitation filter/580-nm emission filter. DAPI nuclear stain was observed with a 359-nm excitation filter/461-nm emission filter. Images were acquired with Zeiss AxioCam MR3 camera operating with Zeiss AxioVision FRET software. Brightness and contrast were adjusted with ImageJ software with identical changes for comparing image sets.

Quantification of OCRL and APPL-1 expression at axon/soma junctions was carried out using ImageJ. Single channels from each image were converted into 8-bit greyscale images and equally adjusted to subtract background. Junctions were manually segmented, and total segment fluorescence was recorded. Segment fluorescence was adjusted based on corrected total cellular fluorescence.

### Analysis of PI(4,5)P2 and WAVE-1 by quantitative immunocytochemistry (ICC)

PI(4,5)P2 was visualized in both NPCs and neurons using ICC. Images were captured using the same parameters for each channel. Random images (5–10) containing ~ 10 cells were captured from each section. The staining intensity was measured using ImageJ software. The background signals were removed using the thresholding tool. Then, the pixel intensities were measured, and the mean pixel intensity from each image was recorded. The signal was normalized to DAPI (LS samples and siblings) or cyclophilin (690C and 690KO). Statistical analysis of the normalized PI(4,5)P2 levels was determined using the Student’s *t* test. A similar analysis was carried out for WAVE-1 in NPCs. All *p* values were two-tailed.

### Antibodies

**Table:** 

Antibody	Company	Catalogue #	Dilution
Anti-OCRL	Proteintech Group	17695-1-AP	WB, 1:200, ICC, 1:50
Anti-APPL1	OriGene	TA807768	1:500
Phalloidin:FITC	ECM Biosciences	PF7501	1:200
Anti-Actin	ECM Biosciences	AM2021	1:100
Anti-Wave1	Abcam	AB50356	WB, 1:2000; ICC, 1:200
Anti-TGN46	Sigma-Aldrich	SAB4200355	1:100
α-Fetoprotein	R&D Systems	MAB1369	1:50
α-Smooth Actin	R&D Systems	MAB1420	1:50
β-III Tubulin	R&D Systems	MAB1195	1:50

### PCR primers used in this study

**Table:** 

Gene	Forward	Reverse
β2M	gctcgcgctactctctcttt	caatgtcggatggatgaaac
OCRL_LS500	cctgcatgaccagaatttga	ttaaaagcgctatgctgacg
OCRLexp-F	acaggtcctgcttcccacta	tggaggtggatgtctaggca
OCRLexp2-F	atccacctccagagcaacac	gctgtgggaaggagcaatag
OCRL_KO	agagctgccctcatttcctt	tgggcctggacttgataaaa
LS100cDNA	ttttcttggaagccctgcca	tgccataaggttgggtggag
LS300cDNA	agcgtcaatgccaacatgatc	aaggagggattaggaaacgctc
OCRL_LS100/300	attgtgttggccatgaggag	ggaggcctcaggagaagact

### Sequencing primers

**Table:** 

LS100seq	aatactcttagtgcattgtatc
LS300seq	tagaagttagacagatgaaatg

## Results

Patient-specific iPS cell lines were developed from three boys with LS and their typically developing male siblings. LS100 is a 17-year-old boy who was delivered vaginally after a 36-week gestation. His mother was 34 years old at the time. Routine maternal alpha-fetoprotein (AFP) testing during pregnancy revealed a marked elevation, which resulted in an amniotic fluid analysis that also showed an increase. Ultrasound in the third trimester showed an excessive accumulation of amniotic fluid (polyhydramnios). At birth, mild hypotonia was noted. At the first postnatal visit, bilateral cataracts were detected, which were removed at 5 and 7 weeks. LS was suspected and confirmed at 10 months of age with an inositol polyphosphate 5-phosphatase OCRL-1 activity assay on fibroblasts (0.54 nmol/min/mg protein [normal controls were 8.18 and 10.10]). The child had delayed developmental milestones; he walked at 2 years, used single words at 2, and put together a string of a couple of words at age 3. Early educational intervention was begun in preschool, and the boy was educated in a regular school in a self-contained classroom. He was able to read individual words at age 5. From the ages of 3–7, he suffered from periodic grand mal seizures, which were controlled with medication. He was treated with human growth hormone because of short stature and hypotonia. He is currently being treated for renal tubular acidosis. The patient was diagnosed with OCD, which is well-controlled with escitalopram. LS200 is his typically developing older brother who was 22 years old when blood was provided to generate iPS cells.

LS300 is a 19-year-old boy diagnosed in infancy. He was a full-term breach delivered by C-section to his 29-year-old mother. Routine AFP screening of maternal blood during pregnancy showed elevated levels, which was confirmed by amniocentesis. He was born with severe hypotonia and bilateral cataracts, which were removed at 4 months. He contracted whooping cough only 2 weeks after birth. LS was diagnosed by analyzing skin fibroblasts for OCRL enzyme activity (results not available). There was a delay in major milestones. He walked at 2.5 years and spoke words at age 3. There was an episode of seizures at age 18, perhaps precipitated by a febrile illness, but subsequently, he has been free of seizure activity without medication. The patient has renal tubular acidosis, scoliosis and hypotonia, and is being treated with escitalopram for OCD and anxiety. LS400 is his typically developing older brother who was 21 years old when he provided blood for iPS cell development.

LS500 is a 9-year-old boy who was delivered vaginally at full term. His mother was 36-years-old. Cataracts were observed at birth, along with hypotonia. Cataracts were removed at 4 weeks, and a diagnosis of LS was made at ~ 8 weeks based on clinical features, which was confirmed by DNA analysis. The child walked at 3.5 years and began to talk at around the same time. There is no history of epilepsy. Renal tubular acidosis, hypotonia, and glaucoma are the primary persistent features. He is being educated in a regular public school with additional support and is able to read two grade levels below his chronological age. Cognitive function was assessed at age 8 using the Wechsler Intelligence Scale for Children—Fifth Edition (WISCV), which provides composite scores that represent intellectual functioning in specified cognitive domains (i.e., verbal comprehension, visual spatial, fluid reasoning, working memory, and processing speed). Each domain was below the fifth percentile, and the full-scale IQ (FSIQ) composite score was 65 (first percentile). LS600 is his typically developing older brother (age 13. iPS cells were also generated from another typically developing male siblihg (age 16), which has not yet been analyzed).

### Analysis of patient-specific mutations

**Fig. 1:**
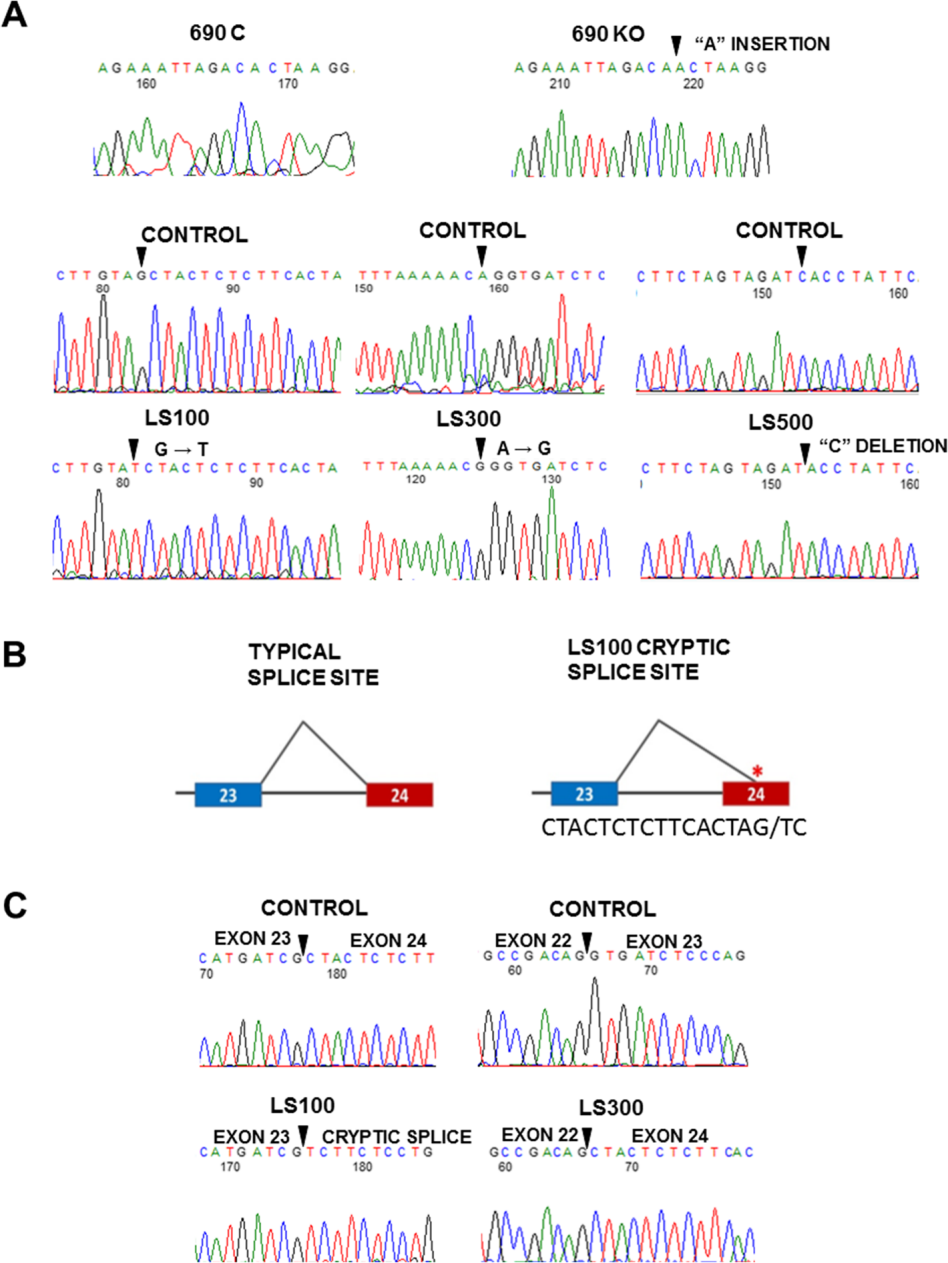
DNA and cDNA sequencing. **a** Genomic DNA sequences showing mutations in the CRISPR-engineered knockout line (690KO) and the LS samples (LS100, LS300, and LS500) along with controls. The arrows point to the mutations. **b** The LS100 splice acceptor mutation predicts the loss of the natural splice site at the intron 23/exon 24 border, as well as a cryptic splice site 16 bases into exon 24. **c** cDNA sequencing showing normal exon 22/23 and exon 23/24 combinations in controls, and aberrant splicing in LS300, which leads to the exclusion of exon 23, thereby connecting exon 22 to 24; and the cryptic splice in LS100, as predicted in panel **b**

To identify the LS-causing mutations, DNA was sequenced at each OCRL exon and intron-exon junction by GeneDx (Gaithersburg, MD); disease-causing mutations were found in all three patient samples, which were confirmed and characterized in our lab. LS100 was found to have a G>T transversion at the canonical “AG” splice acceptor site in intron 23, one base pair upstream from cDNA position 2582 (c.2582-1 G>T). LS300 was found to have a canonical splice acceptor mutation in intron 22, an A>G transition two bases upstream from cDNA position 2470 (c.2470-2 A>G) (Fig. 1a). Finally, LS500 was found to have a “C” deletion in exon 20 at codon 727 (ChrX:128721069 [hg19]), which is cDNA position 2179 (2179delC). This leads to a predicted frame shift and a premature termination signal after 80 codons.

In order to understand how the LS100 and LS300 canonical splice acceptor mutations influence splicing, we sequenced cDNA using primers in the exons flanking the mutations. As seen in Fig. 1c, the control sample shows the expected connection between exons 22 and 23. By contrast, cDNA sequencing of LS300 shows that exon 22 is joined to exon 24; exon 23 is completely bypassed. The loss of exon 23 disrupts the reading frame in exon 24 and leads to the predicted loss of 78 amino acids in the C-terminal end, which contains the RhoGAP domain. Since the cDNA sequence strip shows no evidence of a mixture of mutant and normal splice variants, which would manifest as superimposed, out of frame bases and a chaotic sequencing strip following the frameshift, the c.2470-2 A>G splice site mutation results in the complete absence of normal splicing at exon 23.

The LS100 splice acceptor mutation is interesting. The mutation predicts that the intron 23/exon 24 splice acceptor site will not be recognized (AG/CT>AT/CT). In addition, the first 16 bases of exon 24 resembles a splice acceptor site (CTACTCTCTTCACTAG/TC), with an “AG” preceded by a putative polypyrimidine tract, which suggests that a cryptic slice site might be generated if the natural intron 23/exon24 slice site is bypassed (Fig. 1b). Indeed, as seen in Fig. 1c, the normal joining of exons 23 and 24 seen in the cDNA sequence of the control sample does not occur, and the predicted cryptic splice site in exon 24 is observed. This leads to the loss of 16 bases from the mRNA and a frame shift resulting in the addition of three novel amino acids followed by a premature termination codon, and the loss of 41 amino acids from the OCRL C-terminal end.

Finally, we engineered an *OCRL* null allele using CRISPR-Cas9 gene editing, targeting exon 6 (see “Methods”). This resulted in several lines, which contained in-frame deletions that have not been further characterized. One line with a null variant was recovered, 690KO, which contains an “A” nucleotide insert at codon 137 in exon 6 (position chrX:128691900 [hg19]; cDNA position 413) (Fig. 1a) that results in a predicted frame shift leading to premature termination after one codon.

**Fig. 2:**
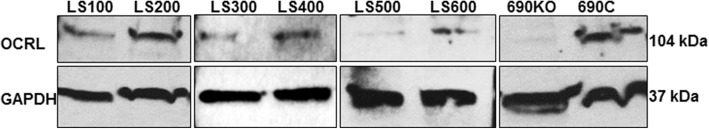
Western blot showing OCRL and GAPDH proteins in NPCs. GAPDH was used as a loading control

Western blotting was carried out to examine OCRL protein and to establish that the 690KO frameshift mutation resulted in the loss of OCRL protein expression. As seen in Fig. 2, no protein was detected in NPCs derived from the KO line, whereas it is expressed in the isogenic control parent line. OCRL protein is detected in the patient-derived LS samples. Although the mutant OCRL proteins are predicted to be somewhat smaller than the normal protein, the difference is not sufficient to be resolved on a 12% polyacrylamide for a protein as large as OCRL (104 kDa).

### OCRL expression in NPCs and early differentiating neurons

**Fig. 3:**
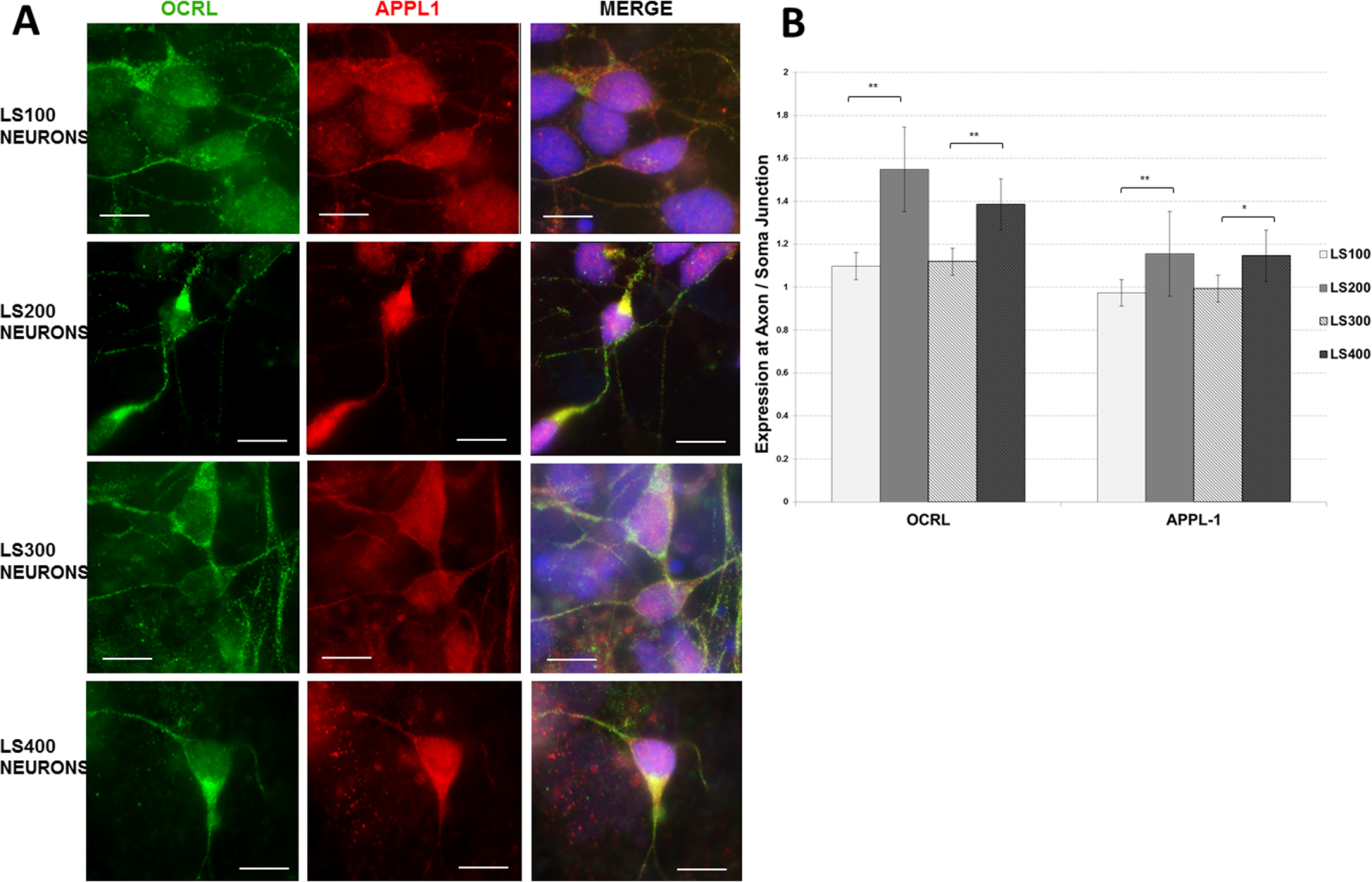
**a**. OCRL and APPL-1 immunocytochemistry showing hyper-dense localization in control neuronal samples (LS200 and LS400) (arrows) compared with patient samples (LS100 and LS300). Neuronal differentiation was carried out once for each sample. All images were captured with the × 63 objective. A 10-μm bar is shown as a reference size marker. **b**. The immunofluorescent signals were quantified for the same area at a total of 66 soma/axon junctions for OCRL and 90 for APPL1. Differences in signal intensity were assessed between the LS samples vs their corresponding sibling controls and analyzed using a Student’s *t* test. One asterisk denotes a two-tailed *p* value of < 0.05; two asterisks indicate a *p* value of < 0.01 (actual values are OCRL: LS100 vs LS200 [1.35E-05]; LS300 vs LS400 [8.03E-04]. APPL1: LS100 vs LS200 [3.91E-05; LS300 vs LS400 [1.84E-02]). The error bars show the standard error of the mean

Immunocytochemistry (ICC) was used to assess the intracellular OCRL expression pattern in early differentiating neurons derived from iPS cells. As seen in Fig. 3, there is an asymmetric pattern of OCRL immunoreactivity in control samples (LS200 and LS400) in early differentiating neurons (day 14 after differentiation). A diffuse, low-level expression pattern is seen throughout the soma, along with a hyper-dense staining pattern occurring primarily at the junction between the soma and projections. The asymmetric pattern of expression suggests that OCRL may be involved in neuronal polarization and/or neurite outgrowth. It is interesting to note in this regard that depletion of OCRL in epithelial cells results in a failure to polarize apical markers [45].

In addition, a punctate pattern is detected in neuronal projections suggesting that OCRL could be involved in the axonal transport system that shuttles endosomes and other cargo to and from the soma and presynaptic terminal, a hypothesis that is currently under investigation.

By contrast, in patient samples (LS100 and LS300), OCRL immunoreactivity is more sparse compared with controls at the hyper-dense regions. We quantified the OCRL signal at multiple soma/axon junctions in two LS samples and their sibling controls. As seen in Fig. 3a, some soma/axon junctions in the control samples show intense OCRL immunoreactivity; most are more modest. Overall, there was a statistically significant 41% decrease in OCRL immunoreactivity at soma/axon junctions in LS100 compared with LS200 (Student’s *t* test, *p* value = 1.35E−05) and a 23% decrease in LS300 vs LS400 (*p* value = 8.03E−04) (Fig. 3b). The immunofluorescent signals were quantified for the same area at a total of 66 soma/axon junctions for OCRL and 90 for APPL1 in a single neuronal induction for each set.

As noted above, *OCRL* mutations affecting the ASH-RhoGAP domain have been found to attenuate the interaction between OCRL and the endosome adaptor protein APPL1 in Cos-7 cells [7]. As seen in the control samples, APPL1 has a diffuse staining pattern similar to OCRL and accumulates in the hyper-dense regions of OCRL immunoreactivity. Furthermore, similar to OCRL, the expression level is lower in those regions in the LS samples. Overall, a statistically significant ~ 19% decrease was seen in LS100 and a ~ 15% decrease in LS300 compared to their controls (*p* value = 3.91E−05; *p* value = 1.84E−02, respectively. The pattern appears to be somewhat different from the findings described by McCrea et al. [7] in Cos-7 cells. In that study, APPL1-positive/OCRL-negative puncta were seen in some regions, consistent with the idea that APPL1 recruits OCRL to those areas. If the same mechanism was occurring in the hyper-dense regions, one would expect APPL1 accumulation in those regions to be the same in control and LS neurons. Our observation is more consistent with the idea that OCRL is drawn to these regions in an APPL1-independent manner, after which APPL1 is being recruited.

### F-actin and WAVE-1 expression is altered in LS neuronal cells

OCRL is an important regulator of actin remodeling and cytoskeletal dynamics. In kidney cell lines, OCRL deficiency leads to the accumulation of PI(4,5)P2 in early endosomes, which induces N-WASP (Wiskott-Aldrich syndrome protein)-dependent increases in endosomal F-actin [6] and an increase in actin comets in endosomes [46], perhaps due to the presence of PI(4,5)P2 on intercellular bridges, which would lead to an increase in actin polymerization. However, in fibroblasts from patients with LS and Dent-2 disease, a decrease in actin stress fibers in fibroblasts has been observed [47, 48].

**Fig. 4:**
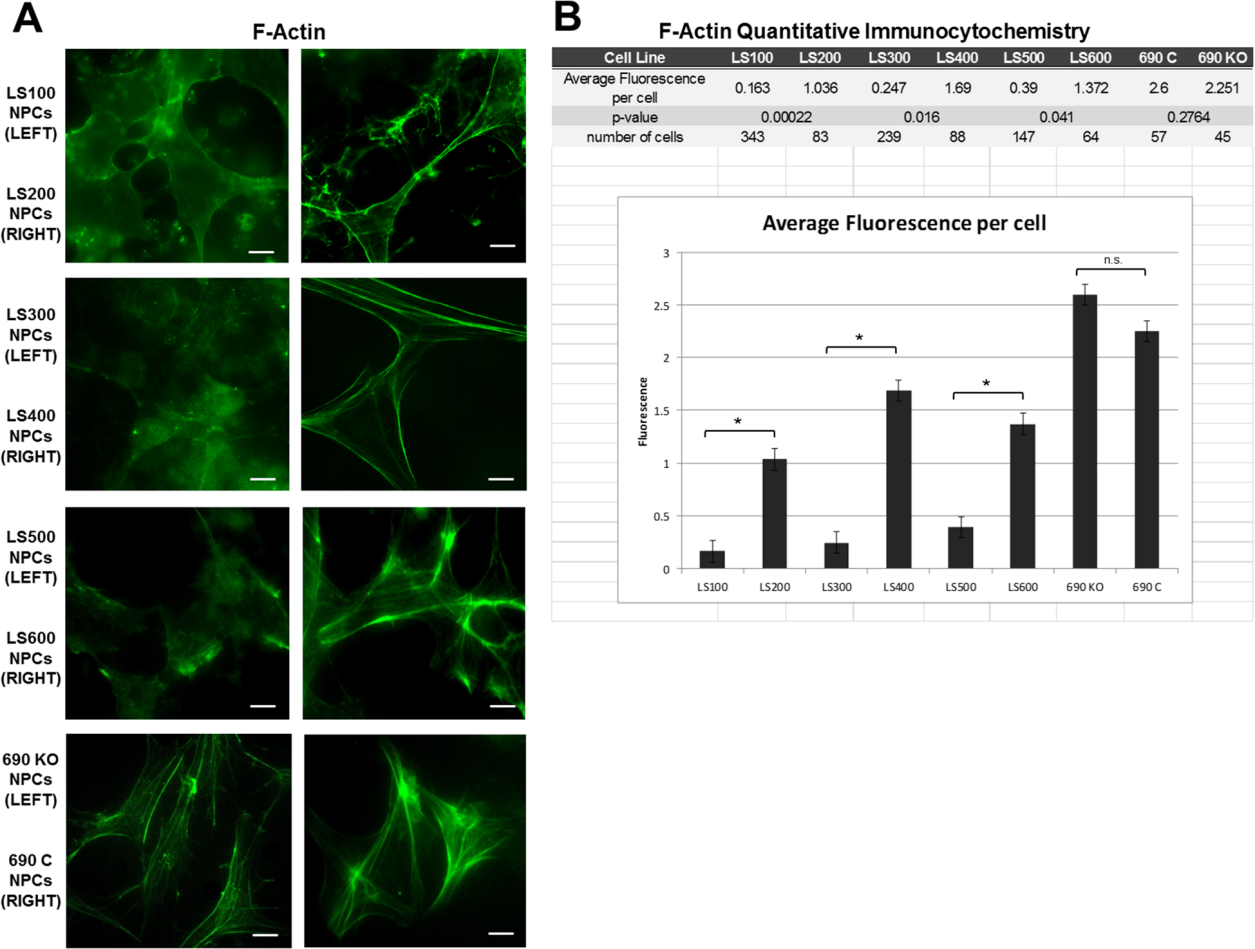
Immunocytochemistry showing F-actin expression in NPCs. **a**. F-actin staining pattern in control NPCs (LS200, LS400, and LS600) nicely outlines the cytoskeleton, while the expression pattern in the patient samples (LS100, LS300, and LS500) is amorphous. Cytoskeletal outline is seen in both 690C and 690KO NPCs. F-actin staining pattern observed in duplicate samples. **b**. F-actin expression by quantitative immunocytochemistry. Asterisk indicates *p* < 0.05 using Student’s *t* test, two-tailed. Error bars are standard deviations

As seen in Fig. 4a, the NPCs from controls show a dense F-actin staining pattern with prominent fibers outlining the cytoskeleton. By contrast, the LS NPCs have a more diffuse pattern and an apparent decrease in signal intensity. This staining pattern was observed in duplicate NPC samples.

Quantitative ICC showed a significant, several-fold decrease in F-actin immunoreactivity (Fig. 4b). We also analyzed the F-actin expression pattern in the CRISPR/Cas9 engineered line (690KO) line, along with its isogenic control (690C). Interestingly, the F-actin staining pattern in control and KO NPCs is quite similar to each other; both show dense fibers, and no differences were detected between the two following quantitative ICC (Fig. 4b).

The findings suggest that the abnormal expression pattern seen in the LS samples, which express hypomorphic *OCRL* variants, is not recapitulated in *OCRL* null NPCs.

**Fig. 5:**
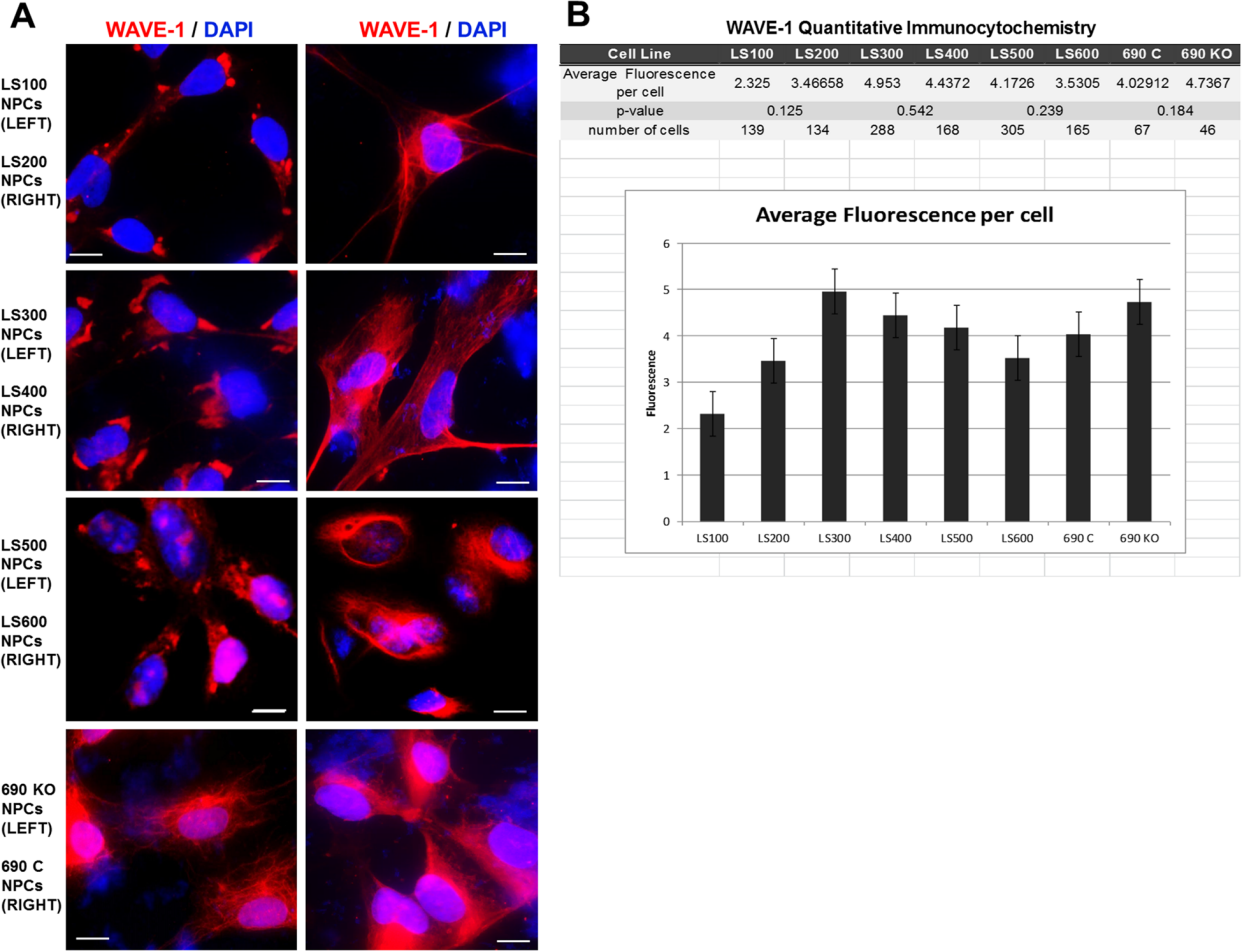
**a**. Immunocytochemistry showing WAVE-1 expression pattern in NPCs. The control samples (LS200, 400 and 600; 690C) show a robust staining pattern with localization to the cytoskeleton. In the patient samples (LS100, 300 and 500), expression is patchy and large inclusions are seen. Inclusions are not seen in the 690KO line. The WAVE-1 inclusions were observed in duplicate NPC samples. **b**. WAVE-1 expression by quantitative immunocytochemistry. Differences between control and patient samples were not statistically significant using Student’s *t* test, two-tailed. Error bars are standard deviations

A key regulator of actin polymerization is the WAVE regulatory complex (WRC), which initiates F-actin nucleation through an interaction with the Arp2/3 complex [49–52]. One of the components of WRC is the Wiskott-Aldrich syndrome protein that includes several family members, one of which, WAVE-1, is expressed at high levels in the brain compared to other cells/tissue [53]. Consequently, we analyzed WAVE-1 by ICC. As seen in Fig. 5a, there is a marked difference in the pattern of WAVE-1 immunoreactivity in LS and control NPCs. In the control samples, the WAVE-1 expression pattern is similar to the F-actin pattern, with a robust, dense expression pattern. However, in the LS samples, this is not seen. Instead, there is a more disorganized expression pattern with large, patchy accumulations of WAVE-1. This was observed in duplicate NPC samples.

Quantitative ICC was used to quantify the WAVE-1 signal. No differences in the sibling sets were detected (Fig. 5b). Thus, the observed differences in WAVE-1 expression are qualitative rather than quantitative.

We also analyzed the WAVE-1 expression pattern in 690KO and 690C NPCs. Interestingly, similar to the F-actin findings, 690 and 690C NPCs show the same, robust WAVE-1 staining pattern similar to the control NPCs (LS200, LS400, LS600); the patchy inclusions seen in the LS samples are not observed in 690KO NPCs. This supports the idea that hypomorphic *OCRL* variants have a phenotype in NPCs that is not recapitulated in *OCRL* null NPCs. The F-actin and WAVE-1 findings were observed in replicate NPC samples.

**Fig. 6:**
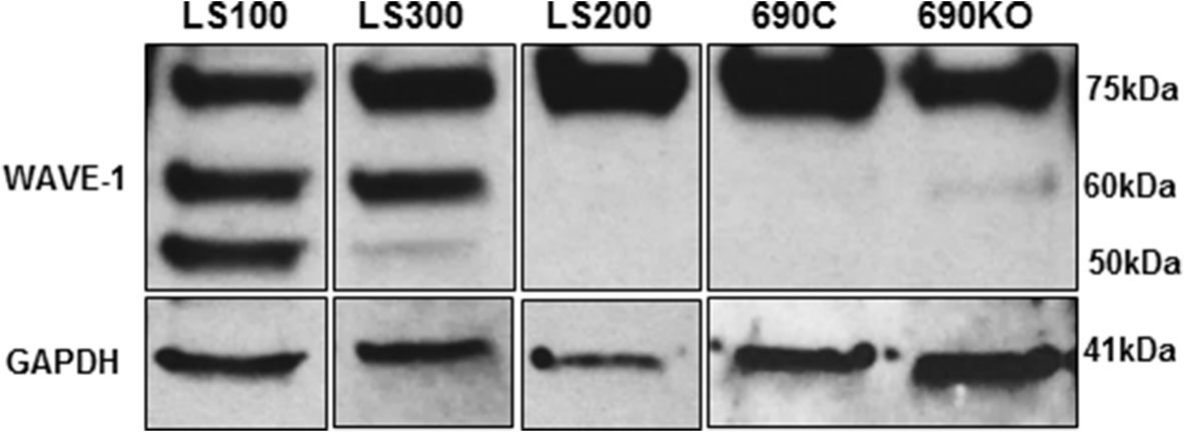
WAVE-1 Western blot. Total cellular protein lysates from neurons derived from patient lines (LS100 and LS300), along with controls and the 690KO line. GAPDH was used as a loading control. The predicted WAVE-1 protein band at ~ 75 kDa is seen in all samples. However, two low molecular weight bands are seen in the LS100 and LS300 samples, which are not seen in controls. One of these bands is barely visible in the 690KO sample. WAVE-1 quantification

WAVE-1 was also analyzed by Western blotting (WB); a marked difference was seen in the LS samples compared to controls (Fig. 6). In the control samples, a single band was observed at ~ 75 kDa in both. However, in the two LS neuronal samples, two lower molecular weight bands were detected. The nature of these bands is not known at this time, but we speculate that they represent WAVE-1 breakdown products. Similar to the findings shown in Fig. 5, there was no quantitative difference in WAVE-1 expression between the control and patient samples.

By contrast, the 690 KO line resembled the control samples and only showed a weak signal corresponding to the presumed degradation product. Thus, similar to the ICC analysis, WAVE-1 expression in the 690KO line resembles controls rather than the LS samples.

Overall, the F-actin and WAVE-1 findings suggest that similar to the mouse *Ocrl* KO, the complete absence of OCRL protein may be less detrimental than expressing a hypomorphic protein (see “Discussion”).

### PI(4,5)P2 expression is increased in OCRL-deficient NPCs

**Fig. 7:**
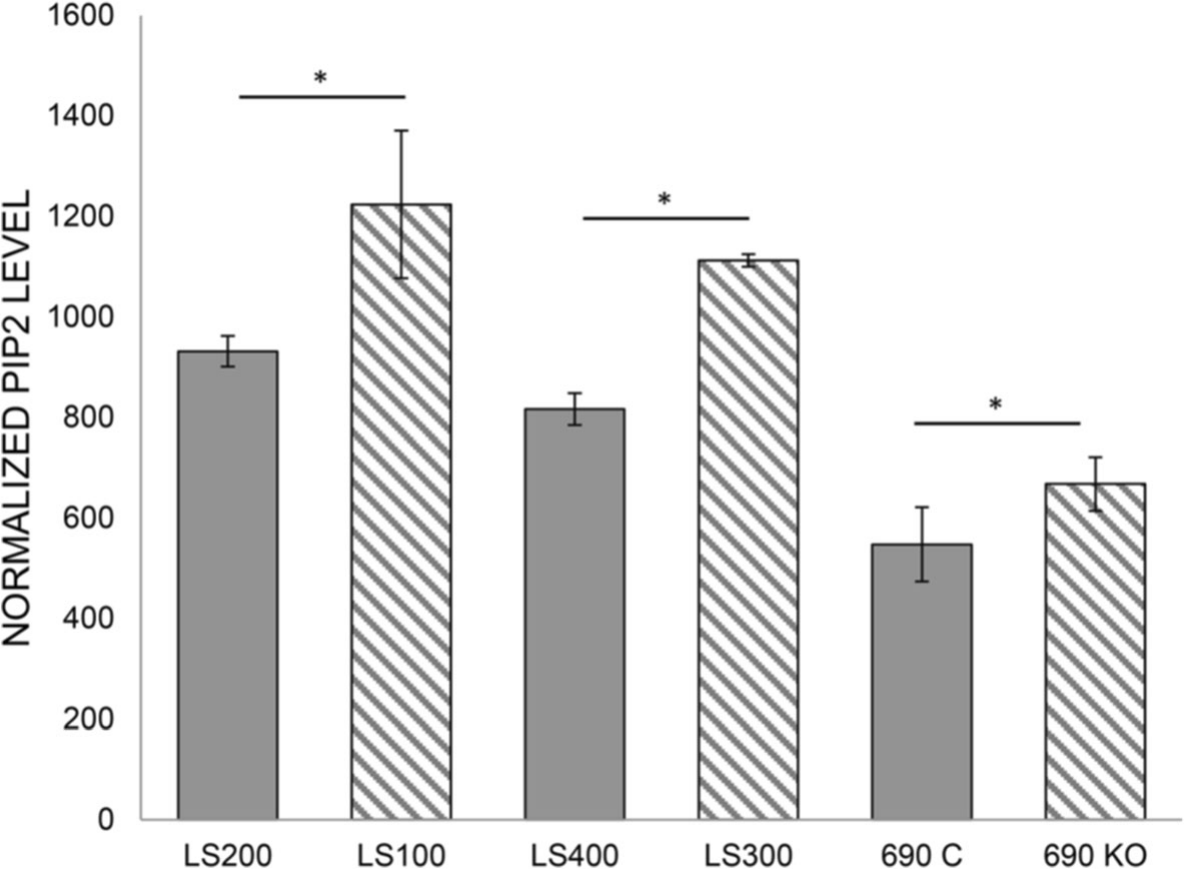
PIP2 levels in neuronal cells by quantitative ICC. The relative level of PIP2 was normalized against a nuclear marker. The LS100/LS200 set was carried out on NPCs, while the analysis of the LS300/LS400 and 690KO/690C sets were carried out on day 14 neurons. The asterisk denotes statistical significance using the Student’s *t* test, two-tailed: LS100 and LS200; *p* = 0.025, LS300 and LS400; *p* = 0.003, 690C and 690KO; *p* = 0.0001. Error bars are standard deviations. A single NPC or neuronal induction was carried out for each set, with between 88 and 128 cells quantified for each

Since PI(4,5)P2 activates WRC and is a major OCRL substrate, we measured its relative level of expression by quantitative ICC by normalizing its expression in NPCs against a nuclear marker. As seen in Fig. 7, there was a statistically significant, ~ 25% increase in PI(4,5)P2 levels in the patient samples compared with their unaffected siblings, as one would predict in OCRL-deficient cells. A significant increase was also seen in the KO line compared to its control. Thus, unlike the WAVE-1 findings, which are seen in the LS samples but not in 690KO, the increase in PI(4,5)P2 is similar in all OCRL-deficient NPCs and neurons. Between 88 and 128 cells were quantified for each sample in a single neuronal/NPC induction.

The finding supports the hypothesis that in the complete absence of OCRL, some cellular and molecular phenotypes can be rescued, but others are not. This is consistent with the clinical manifestations of some Dent-2 patients who have null mutations in the 5′ end of the gene who do have renal disease, but not neurodevelopmental problems. The findings in the patient samples also suggest that in neuronal cells, there is an inverse relationship between PI(4,5)P2 and F-actin/WAVE-1 dynamics, which is opposite the findings in kidney cell lines [6, 7], but similar to findings in fibroblasts from LS patients [47, 48].

## Discussion

We have developed an iPS cell model for Lowe syndrome, which provides an opportunity to grow patient-specific neurons in vitro. Based on studies carried out on non-neuronal cells over the years, OCRL deficiency causes deficits in endosome recycling and transport, and endosomal actin dynamics. In this initial report, we were particularly interested in determining whether LS mutations affect F-actin polymerization in neuronal cells since this is a key regulator of neuronal migration, neurite outgrowth, dendritic spine formation and NMDA and AMPA receptor recycling, and defects in these phenomena have been found in many different genetic subgroups of SZ, ASD, and intellectual and developmental disabilities (IDD) [54–64]. Most studies show a decrease or disruption of F-actin expression, similar to our findings in LS neuronal cells. However, an increase in F-actin polymerization has been found in Fragile X [65]. Thus, altered F-actin dynamics—either increases or decreases in expression and structure—can lead to neurodevelopmental problems.

In the lines derived from LS patients, we found abnormalities in the expression of F-actin and WAVE-1 in neuronal cells. A decrease in the cytoskeletal organization of both was found. This is different from the effects of OCRL deficiency in other cell types where an increase in endosomal F-actin has been observed [6, 7]. This effect is likely due to an increase in endosomal PI(4,5)P2, which induces F-actin recruitment through the activation of N-WASP [6, 66]. However, in fibroblasts from patients with OCRL and Dent-2 disease, a decrease in actin stress fibers has been observed [47, 48]. This effect was not correlated with PI(4,5)P2 levels, similar to our findings, which show an inverse relationship between F-actin and PI(4,5)P2 in LS neuronal cells.

The disorganized expression of WAVE-1 was characterized by the formation of large inclusions, the cause of which is currently under investigation. One possibility being explored is the involvement of the autophagy-lysosome pathway. Recently, De Leo et al. showed that OCRL regulates autophagosome-lysosome fusion in HK-2 cells [67].

WAVE-1 is one of five proteins that constitute the WRC in neuronal cells, which include CYFIP1, ABI2, Nap1, and HSPC300 (BRK1) or their orthologs [68–70]. Consistent with the association between defective F-actin polymerization and neurodevelopmental disorders, several genes coding for WRC components have also been implicated in these disorders. For example, CYFIP1, which is an FMR1 interacting protein 1, plays a role in synaptic function and appears to be the gene responsible for 15q11.2-related neurodevelopmental disorders [56, 60, 69, 71, 72]. Further, shRNA-mediated knockdown (KD) of CYFIP1 leads to a significant decrease in WAVE-1 and F-actin expression in NPCs derived from iPS cells [56].

In addition, rare variants in *ABI2* have been found in an exome sequencing study in consanguineous families with intellectual disabilities [73]. Also, *NCKAP1*, which codes for a WAVE-1 regulatory protein, has been identified as a strong candidate gene for ASD and IDD [74, 75].

The disconnect we observe between PI(4,5)P2 expression with F-actin and WAVE-1 organization suggests that another aspect of OCRL deficiency, aside from its effect as a PI(4,5)P2 phosphatase, is responsible for the abnormalities we observed. An effect on Rac1, a Rho GTPase, is a possibility. Rac1 activates the WRC in the Arp2/3-mediated polymerization of actin [76, 77] (Additional file 1: Figure S1). Rac1 is an OCRL target; downregulation of OCRL has been found to cause aberrant activation of Rac1 in human chondrocytes [78]. This is likely due to the loss of the GTPase activating domain, which is found in the C-terminal end of OCRL. A reduction in Rac1 GTPase activity could conceivably cause constitutive Rac1 activation because of a reduction in the conversion of GTP (which is bound to the active form of Rac1), to GDP (which leads to Rac1 inactivation). The mutations in LS100, LS300, and LS500 all disrupt the RhoGAP domain. On the other hand, in LS fibroblasts, OCRL deficiency causes a reduction in active Rac1 [79], which would be consistent with the effect we observed on F-actin/WAVE-1 dynamics in LS neuronal cells. Clarifying the role of Rac1 in OCRL-deficient NPCs and neurons is currently under investigation.

It is important to note that Rac1 is dysregulated in certain neurodevelopmental disorders through an effect on synaptic plasticity and dendritic spines [80]. In addition, Rac1 has been found to be upregulated in fragile X syndrome [81, 82], and rare genetic variants in Rac1 regulators have been found in ASD, BD and SZ [57, 83, 84].

It is also interesting to note in the context of F-actin/WAVE-1 regulation that the expression of Arp2/3 complex subunits is significantly downregulated in the prefrontal cortex in SZ postmortem samples [85], and that DISC1 regulates Rac1 activation in response to NMDA receptor stimulation [62].

In summary, our findings suggest that LS, an extremely rare condition, is part of a larger functional subgroup of neurodevelopmental disorders that are caused by mutations that disrupt F-actin/WAVE-1 dynamics. How this disruption affects LS NPC and neuronal function is currently under investigation.

Considering the widespread effects of F-actin polymerization, altered neuronal migration, neurite branching and outgrowth, and dendritic spine formation and function are possible; this too is currently under investigation. However, it is also important to note that non-neuronal cells in the brain—specifically microglia and astrocytes—also require intact F-actin dynamics in their maintenance of synaptic function, which are accomplished by F-actin-dependent processes (e.g., migration and phagocytosis). The effect of abnormal F-actin formation on these cell types in LS and other genetic subgroups of neurodevelopmental disorders can now be studied in vitro using newly defined methods for converting iPS cells into functional astrocytes and microglia [86–88].

Finally, our preliminary findings show some differences between LS neuronal cells and those derived from an *OCRL* KO line we generated using CRISPR-Cas9. This is similar to the absence of an observable phenotype in the mouse *Ocrl* KO model, which has been attributed to rescue by the *OCRL* paralog, *INPP5B* [20]. It is also similar to the absence of a severe neurodevelopmental phenotype in a few patients with Dent-2 disease who have apparent null variants in the 5′ end of *OCRL* [14]. Although additional *OCRL* KO iPS cell lines are currently being generated to confirm our findings, our preliminary observations suggest that hypomorphic variants that produce dysfunctional OCRL proteins may cause more dramatic molecular and cellular alterations than those occurring in the complete absence of OCRL protein, perhaps by competitive inhibition at critical binding sites between dysfunctional OCRL proteins and INPP5B or another compensatory pathway. Whether our findings in KO neuronal cells extend to other cell types relevant to LS pathogenesis, such as renal tubules, remains to be seen. However, the finding that neurodevelopmental problems occur in some patients with OCRL deletions and null variants suggests that compensatory rescue in humans is not universal, owing perhaps to genetic background or polymorphic variation in *INPP5B.* Nevertheless, if our findings are replicated, it would suggest that in some individuals, completely blocking mutant OCRL protein might, paradoxically, have a positive therapeutic effect. The findings also suggest that a more effective approach for generating a mouse LS model would be to “knock in” a hypomorphic variant.

## Conclusion

We have established an iPS cell model for Lowe syndrome, a rare X-linked disorders caused by loss of function mutations in *OCRL*, which codes for inositol polyphosphate 5-phosphatase, a regulator of endosome recycling and actin dynamics. In neuronal cells derived from patient-specific iPS cells, abnormalities were found in the formation of F-actin and WAVE-1, a component of the wave regulatory complex. This property is shared with other ASD and IDD candidate genes. Thus, Lowe syndrome, a rare cause of IDD, is part of a larger subgroup of patients who have a common underlying pathogenic process.

## Additional file

Additional file 1: **Figure S1.** WAVE-1 regulatory complex. Adapted from ref. 89. (TIF 89 kb)

## Abbreviations

AFP: Alpha-feto protein
ASD: Autism spectrum disorders
BD: Bipolar disorder
F-actin: Filamentous actin
FSIQ: Full-scale IQ
ICC: Immunocytochemistry
IDD: Intellectual and developmental disabilities
iPS: Induced pluripotent stem cell
IRB: Institutional Review Board
KD: Knockdown
KO: knockout
LS: Lowe syndrome
NPC: neural progenitor cell
N-WASP: Wiskott-Aldrich syndrome protein
PI(4,5)P2: Phosphatidylinositol 4,5-bisphosphate
SZ: Schizophrenia
WB: Western blotting
WRC: WAVE regulatory complex

## References

[1] Silver DN, Lewis RA, Nussbaum RL (1987). Mapping the Lowe oculocerebrorenal syndrome to Xq24-q26 by use of restriction fragment length polymorphisms. J Clin Invest.

[2] Schurman SJ, Scheinman SJ (2009). Inherited cerebrorenal syndromes. Nat Rev Nephrol.

[3] Waugh MG (2015). PIPs in neurological diseases. Biochim Biophys Acta.

[4] Staiano L, De Leo MG, Persico M, De Matteis MA (2015). Mendelian disorders of PI metabolizing enzymes. Biochim Biophys Acta.

[5] Lewis RA, Nussbaum RL, Brewer ED. Lowe Syndrome. In: Adam MP, Ardinger HH, Pagon RA, Wallace SE, Bean LJH, Stephens K, Amemiya A, editors. Seattle: University of Washington; 1993. 20301653

[6] Vicinanza M, Di Campli A, Polishchuk E, Santoro M, Di Tullio G, Godi A, Levtchenko E, De Leo MG, Polishchuk R, Sandoval L, Marzolo MP, De Matteis MA (2011). OCRL controls trafficking through early endosomes via PtdIns4,5P(2)-dependent regulation of endosomal actin. EMBO J.

[7] McCrea HJ, Paradise S, Tomasini L, Addis M, Melis MA, De Matteis MA, De Camilli P (2008). All known patient mutations in the ASH-RhoGAP domains of OCRL affect targeting and APPL1 binding. Biochem Biophys Res Commun.

[8] Hoopes RR, Shrimpton AE, Knohl SJ, Hueber P, Hoppe B, Matyus J, Simckes A, Tasic V, Toenshoff B, Suchy SF, Nussbaum RL, Scheinman SJ (2005). Dent disease with mutations in OCRL1. Am J Hum Genet.

[9] Nakatsu F, Messa M, Nandez R, Czapla H, Zou Y, Strittmatter SM, De Camilli P (2015). Sac2/INPP5F is an inositol 4-phosphatase that functions in the endocytic pathway. J Cell Biol.

[10] Zhang X, Jefferson AB, Auethavekiat V, Majerus PW (1995). The protein deficient in Lowe syndrome is a phosphatidylinositol-4,5-bisphosphate 5-phosphatase. Proc Natl Acad Sci U S A.

[11] Zhang X, Hartz PA, Philip E, Racusen LC, Majerus PW (1998). Cell lines from kidney proximal tubules of a patient with Lowe syndrome lack OCRL inositol polyphosphate 5-phosphatase and accumulate phosphatidylinositol 4,5-bisphosphate. J Biol Chem.

[12] Pasternack SM, Bockenhauer D, Refke M, Tasic V, Draaken M, Conrad C, Born M, Betz RC, Reutter H, Ludwig M (2013). A premature termination mutation in a patient with Lowe syndrome without congenital cataracts: dropping the "O" in OCRL. Klin Padiatr.

[13] Recker F, Zaniew M, Bockenhauer D, Miglietti N, Bokenkamp A, Moczulska A, Rogowska-Kalisz A, Laube G, Said-Conti V, Kasap-Demir B, Niemirska A, Litwin M, Siten G, Chrzanowska KH, Krajewska-Walasek M, Sethi SK, Tasic V, Anglani F, Addis M, Wasilewska A, Szczepanska M, Pawlaczyk K, Sikora P, Ludwig M (2015). Characterization of 28 novel patients expands the mutational and phenotypic spectrum of Lowe syndrome. Pediatr Nephrol.

[14] Hichri H, Rendu J, Monnier N, Coutton C, Dorseuil O, Poussou RV, Baujat G, Blanchard A, Nobili F, Ranchin B, Remesy M, Salomon R, Satre V, Lunardi J (2011). From Lowe syndrome to Dent disease: correlations between mutations of the OCRL1 gene and clinical and biochemical phenotypes. Hum Mutat.

[15] Utsch B, Bokenkamp A, Benz MR, Besbas N, Dotsch J, Franke I, Frund S, Gok F, Hoppe B, Karle S, Kuwertz-Broking E, Laube G, Neb M, Nuutinen M, Ozaltin F, Rascher W, Ring T, Tasic V, van Wijk JA, Ludwig M (2006). Novel OCRL1 mutations in patients with the phenotype of Dent disease. Am J Kidney Dis.

[16] Watanabe M, Nakagawa R, Kohmoto T, Naruto T, Suga KI, Goji A, Horikawa H, Masuda K, Kagami S, Imoto I (2016). Exome-first approach identified a novel gloss deletion associated with Lowe syndrome. Human Genome Var.

[17] Peces R, Peces C, de Sousa E, Vega C, Selgas R, Nevado J (2013). A novel and de novo deletion in the OCRL1 gene associated with a severe form of Lowe syndrome. Int Urol Nephrol.

[18] Peverall J, Edkins E, Goldblatt J, Murch A (2000). Identification of a novel deletion of the entire OCRL1 gene detected by FISH analysis in a family with Lowe syndrome. Clin Genet.

[19] Addis M, Meloni C, Congiu R, Santaniello S, Emma F, Zuffardi O, Ciccone R, Cao A, Melis MA, Cau M (2007). A novel interstitial deletion in Xq25, identified by array-CGH in a patient with Lowe syndrome. Eur J Med Genetics.

[20] Janne PA, Suchy SF, Bernard D, MacDonald M, Crawley J, Grinberg A, Wynshaw-Boris A, Westphal H, Nussbaum RL (1998). Functional overlap between murine Inpp5b and Ocrl1 may explain why deficiency of the murine ortholog for OCRL1 does not cause Lowe syndrome in mice. J Clin Invest.

[21] Nandez R, Balkin DM, Messa M, Liang L, Paradise S, Czapla H, Hein MY, Duncan JS, Mann M, De Camilli P (2014). A role of OCRL in clathrin-coated pit dynamics and uncoating revealed by studies of Lowe syndrome cells. eLife.

[22] Sharma S, Skowronek A, Erdmann KS (2015). The role of the Lowe syndrome protein OCRL in the endocytic pathway. Biol Chem.

[23] Allmendinger AM, Desai NS, Burke AT, Viswanadhan N, Prabhu S (2014). Neuroimaging and renal ultrasound manifestations of Oculocerebrorenal syndrome of Lowe. J Radiol Case Rep.

[24] Ramirez IB, Pietka G, Jones DR, Divecha N, Alia A, Baraban SC, Hurlstone AF, Lowe M (2012). Impaired neural development in a zebrafish model for Lowe syndrome. Hum Mol Genet.

[25] Bothwell SP, Chan E, Bernardini IM, Kuo YM, Gahl WA, Nussbaum RL (2011). Mouse model for Lowe syndrome/Dent disease 2 renal tubulopathy. J Am Soc Nephrol.

[26] Boland MJ, Nazor KL, Tran HT, Szucs A, Lynch CL, Paredes R, Tassone F, Sanna PP, Hagerman RJ, Loring JF. Molecular analyses of neurogenic defects in a human pluripotent stem cell model of fragile X syndrome. Brain. 2017;140(3):582–98. 10.1093/brain/aww357 PMC5837342 28137726

[27] Dezonne RS, Sartore RC, Nascimento JM, Saia-Cereda VM, Romao LF, Alves-Leon SV, de Souza JM, Martins-de-Souza D, Rehen SK, Gomes FC (2017). Derivation of functional human astrocytes from cerebral organoids. Sci Rep.

[28] Sellgren CM, Sheridan SD, Gracias J, Xuan D, Fu T, Perlis RH (2017). Patient-specific models of microglia-mediated engulfment of synapses and neural progenitors. Mol Psychiatry.

[29] Chandrasekaran A, Avci HX, Leist M, Kobolak J, Dinnyes A (2016). Astrocyte differentiation of human pluripotent stem cells: new tools for neurological disorder research. Front Cell Neurosci.

[30] O'Shea KS, McInnis MG (2016). Neurodevelopmental origins of bipolar disorder: iPSC models. Mol Cell Neurosci.

[31] Mariani J, Coppola G, Zhang P, Abyzov A, Provini L, Tomasini L, Amenduni M, Szekely A, Palejev D, Wilson M, Gerstein M, Grigorenko EL, Chawarska K, Pelphrey KA, Howe JR, Vaccarino FM (2015). FOXG1-dependent dysregulation of GABA/glutamate neuron differentiation in autism spectrum disorders. Cell.

[32] Wang P, Lin M, Pedrosa E, Hrabovsky A, Zhang Z, Guo W, Lachman HM, Zheng D (2015). CRISPR/Cas9-mediated heterozygous knockout of the autism gene CHD8 and characterization of its transcriptional networks in neurodevelopment. Mol Autism.

[33] Lancaster MA, Knoblich JA (2014). Generation of cerebral organoids from human pluripotent stem cells. Nat Protoc.

[34] Yazawa M, Dolmetsch RE (2013). Modeling Timothy syndrome with iPS cells. J Cardiovasc Transl Res.

[35] Lin M, Zhao D, Hrabovsky A, Pedrosa E, Zheng D, Lachman HM (2014). Heat shock alters the expression of schizophrenia and autism candidate genes in an induced pluripotent stem cell model of the human telencephalon. PloS one.

[36] Brennand KJ, Simone A, Jou J, Gelboin-Burkhart C, Tran N, Sangar S, Li Y, Mu Y, Chen G, Yu D, McCarthy S, Sebat J, Gage FH. Modelling schizophrenia using human induced pluripotent stem cells. Nature. 2011;473(7346):221–5. 10.1038/nature09915 PMC3392969 21490598

[37] Tcw J, Wang M, Pimenova AA, Bowles KR, Hartley BJ, Lacin E, Machlovi SI, Abdelaal R, Karch CM, Phatnani H, Slesinger PA, Zhang B, Goate AM, Brennand KJ (2017). An efficient platform for astrocyte differentiation from human induced pluripotent stem cells. Stem Cell Rep.

[38] Zhang Y, Pak C, Han Y, Ahlenius H, Zhang Z, Chanda S, Marro S, Patzke C, Acuna C, Covy J, Xu W, Yang N, Danko T, Chen L, Wernig M, Sudhof TC (2013). Rapid single-step induction of functional neurons from human pluripotent stem cells. Neuron.

[39] Yang N, Chanda S, Marro S, Ng YH, Janas JA, Haag D, Ang CE, Tang Y, Flores Q, Mall M, Wapinski O, Li M, Ahlenius H, Rubenstein JL, Chang HY, Buylla AA, Sudhof TC, Wernig M. Generation of pure GABAergic neurons by transcription factor programming. Nat Methods. 2017;14(6):621–8. 10.1038/nmeth.4291 PMC5567689 28504679

[40] Wilkinson B, Grepo N, Thompson BL, Kim J, Wang K, Evgrafov OV, Lu W, Knowles JA, Campbell DB (2015). The autism-associated gene chromodomain helicase DNA-binding protein 8 (CHD8) regulates noncoding RNAs and autism-related genes. Transl Psychiatry.

[41] Knowles H, Li Y, Perraud AL (2013). The TRPM2 ion channel, an oxidative stress and metabolic sensor regulating innate immunity and inflammation. Immunol Res.

[42] Wang P, Mokhtari R, Pedrosa E, Kirschenbaum M, Bayrak C, Zheng D, Lachman HM (2017). CRISPR/Cas9-mediated heterozygous knockout of the autism gene CHD8 and characterization of its transcriptional networks in cerebral organoids derived from iPS cells. Mol Autism.

[43] Olivier E, Qiu C, Bouhassira EE (2012). Novel, high-yield red blood cell production methods from CD34-positive cells derived from human embryonic stem, yolk sac, fetal liver, cord blood, and peripheral blood. Stem Cells Transl Med.

[44] Ran FA, Hsu PD, Lin CY, Gootenberg JS, Konermann S, Trevino AE, Scott DA, Inoue A, Matoba S, Zhang Y, Zhang F (2013). Double nicking by RNA-guided CRISPR Cas9 for enhanced genome editing specificity. Cell.

[45] Grieve AG, Daniels RD, Sanchez-Heras E, Hayes MJ, Moss SE, Matter K, Lowe M, Levine TP (2011). Lowe syndrome protein OCRL1 supports maturation of polarized epithelial cells. PloS one.

[46] Ueno T, Falkenburger BH, Pohlmeyer C, Inoue T (2011). Triggering actin comets versus membrane ruffles: distinctive effects of phosphoinositides on actin reorganization. Sci Sig.

[47] Montjean R, Aoidi R, Desbois P, Rucci J, Trichet M, Salomon R, Rendu J, Faure J, Lunardi J, Gacon G, Billuart P, Dorseuil O (2015). OCRL-mutated fibroblasts from patients with Dent-2 disease exhibit INPP5B-independent phenotypic variability relatively to Lowe syndrome cells. Hum Mol Genet.

[48] Suchy SF, Nussbaum RL (2002). The deficiency of PIP2 5-phosphatase in Lowe syndrome affects actin polymerization. Am J Hum Genet.

[49] Murk K, Blanco Suarez EM, Cockbill LM, Banks P, Hanley JG (2013). The antagonistic modulation of Arp2/3 activity by N-WASP, WAVE2 and PICK1 defines dynamic changes in astrocyte morphology. J Cell Sci.

[50] Daste F, Walrant A, Holst MR, Gadsby JR, Mason J, Lee JE, Brook D, Mettlen M, Larsson E, Lee SF, Lundmark R, Gallop JL. Control of actin polymerization via the coincidence of phosphoinositides and high membrane curvature. J Cell Biol. 2017;216(11):3745–65. 10.1083/jcb.201704061 PMC5674896 28923975

[51] Chia PH, Chen B, Li P, Rosen MK, Shen K (2014). Local F-actin network links synapse formation and axon branching. Cell.

[52] Alekhina O, Burstein E, Billadeau DD (2017). Cellular functions of WASP family proteins at a glance. J Cell Sci.

[53] Shah K, Rossie S (2018). Tale of the good and the bad Cdk5: remodeling of the actin cytoskeleton in the brain. Mol Neurobiol.

[54] Bralten J, van Hulzen KJ, Martens MB, Galesloot TE, Arias Vasquez A, Kiemeney LA, Buitelaar JK, Muntjewerff JW, Franke B, Poelmans G. Autism spectrum disorders and autistic traits share genetics and biology. Mol Psychiatry. 2018;23(5):1205–12. 10.1038/mp.2017.98 PMC5984081 28507316

[55] Deans PJM, Raval P, Sellers KJ, Gatford NJF, Halai S, Duarte RRR, Shum C, Warre-Cornish K, Kaplun VE, Cocks G, Hill M, Bray NJ, Price J, Srivastava DP (2017). Psychosis risk candidate ZNF804A localizes to synapses and regulates neurite formation and dendritic spine structure. Biol Psychiatry.

[56] Nebel RA, Zhao D, Pedrosa E, Kirschen J, Lachman HM, Zheng D, Abrahams BS (2016). Reduced CYFIP1 in human neural progenitors results in dysregulation of schizophrenia and epilepsy gene networks. PloS one.

[57] Sadybekov A, Tian C, Arnesano C, Katritch V, Herring BE (2017). An autism spectrum disorder-related de novo mutation hotspot discovered in the GEF1 domain of Trio. Nat Commun.

[58] Lilja J, Zacharchenko T, Georgiadou M, Jacquemet G, De Franceschi N, Peuhu E, Hamidi H, Pouwels J, Martens V, Nia FH, Beifuss M, Boeckers T, Kreienkamp HJ, Barsukov IL, Ivaska J (2017). SHANK proteins limit integrin activation by directly interacting with Rap1 and R-Ras. Nat Cell Biol.

[59] Yan Z, Kim E, Datta D, Lewis DA, Soderling SH (2016). Synaptic actin dysregulation, a convergent mechanism of mental disorders?. J Neurosci.

[60] Hsiao K, Harony-Nicolas H, Buxbaum JD, Bozdagi-Gunal O, Benson DL (2016). Cyfip1 regulates presynaptic activity during development. J Neurosci.

[61] Han K, Chen H, Gennarino VA, Richman R, Lu HC, Zoghbi HY (2015). Fragile X-like behaviors and abnormal cortical dendritic spines in cytoplasmic FMR1-interacting protein 2-mutant mice. Hum Mol Genet.

[62] Hayashi-Takagi A, Takaki M, Graziane N, Seshadri S, Murdoch H, Dunlop AJ, Makino Y, Seshadri AJ, Ishizuka K, Srivastava DP, Xie Z, Baraban JM, Houslay MD, Tomoda T, Brandon NJ, Kamiya A, Yan Z, Penzes P, Sawa A (2010). Disrupted-in-schizophrenia 1 (DISC1) regulates spines of the glutamate synapse via Rac1. Nat Neurosci.

[63] Miyoshi K, Honda A, Baba K, Taniguchi M, Oono K, Fujita T, Kuroda S, Katayama T, Tohyama M (2003). Disrupted-in-schizophrenia 1, a candidate gene for schizophrenia, participates in neurite outgrowth. Mol Psychiatry.

[64] Lin YC, Frei JA, Kilander MB, Shen W, Blatt GJ (2016). A subset of autism-associated genes regulate the structural stability of neurons. Front Cell Neurosci.

[65] Pyronneau A, He Q, Hwang JY, Porch M, Contractor A, Zukin RS. Aberrant Rac1-cofilin signaling mediates defects in dendritic spines, synaptic function, and sensory perception in fragile X syndrome. Sci Signal. 2017;10(504) 10.1126/scisignal.aan0852. 10.1126/scisignal.aan0852 PMC5988355 29114038

[66] Saarikangas J, Zhao H, Lappalainen P (2010). Regulation of the actin cytoskeleton-plasma membrane interplay by phosphoinositides. Physiol Rev.

[67] De Leo MG, Staiano L, Vicinanza M, Luciani A, Carissimo A, Mutarelli M, Di Campli A, Polishchuk E, Di Tullio G, Morra V, Levtchenko E, Oltrabella F, Starborg T, Santoro M, Di Bernardo D, Devuyst O, Lowe M, Medina DL, Ballabio A, De Matteis MA (2016). Autophagosome-lysosome fusion triggers a lysosomal response mediated by TLR9 and controlled by OCRL. Nat Cell Biol.

[68] Chen Z, Borek D, Padrick SB, Gomez TS, Metlagel Z, Ismail AM, Umetani J, Billadeau DD, Otwinowski Z, Rosen MK (2010). Structure and control of the actin regulatory WAVE complex. Nature.

[69] Abekhoukh S, Sahin HB, Grossi M, Zongaro S, Maurin T, Madrigal I, Kazue-Sugioka D, Raas-Rothschild A, Doulazmi M, Carrera P, Stachon A, Scherer S, Drula Do Nascimento MR, Trembleau A, Arroyo I, Szatmari P, Smith IM, Mila M, Smith AC, Giangrande A, Caille I, Bardoni B (2017). New insights into the regulatory function of CYFIP1 in the context of WAVE- and FMRP-containing complexes. Dis Model Mech.

[70] Mendoza MC (2013). Phosphoregulation of the WAVE regulatory complex and signal integration. Semin Cell Dev Biol.

[71] Oguro-Ando A, Rosensweig C, Herman E, Nishimura Y, Werling D, Bill BR, Berg JM, Gao F, Coppola G, Abrahams BS, Geschwind DH. Increased CYFIP1 dosage alters cellular and dendritic morphology and dysregulates mTOR. Mol Psychiatry. 2015;20(9):1069–78. 10.1038/mp.2014.124 PMC4409498 25311365

[72] Napoli I, Mercaldo V, Boyl PP, Eleuteri B, Zalfa F, De Rubeis S, Di Marino D, Mohr E, Massimi M, Falconi M, Witke W, Costa-Mattioli M, Sonenberg N, Achsel T, Bagni C (2008). The fragile X syndrome protein represses activity-dependent translation through CYFIP1, a new 4E-BP. Cell.

[73] Harripaul R, Vasli N, Mikhailov A, Rafiq MA, Mittal K, Windpassinger C, Sheikh TI, Noor A, Mahmood H, Downey S, Johnson M, Vleuten K, Bell L, Ilyas M, Khan FS, Khan V, Moradi M, Ayaz M, Naeem F, Heidari A, Ahmed I, Ghadami S, Agha Z, Zeinali S, Qamar R, Mozhdehipanah H, John P, Mir A, Ansar M, French L, Ayub M, Vincent JB. Mapping autosomal recessive intellectual disability: combined microarray and exome sequencing identifies 26 novel candidate genes in 192 consanguineous families. Mol Psychiatry. 2018;23(4):973–84. 10.1038/mp.2017.60 28397838

[74] Freed D, Pevsner J (2016). The contribution of mosaic variants to autism spectrum disorder. PLoS Genetics.

[75] Anazi S, Maddirevula S, Salpietro V, Asi YT, Alsahli S, Alhashem A, Shamseldin HE, AlZahrani F, Patel N, Ibrahim N, Abdulwahab FM, Hashem M, Alhashmi N, Al Murshedi F, Al Kindy A, Alshaer A, Rumayyan A, Al Tala S, Kurdi W, Alsaman A, Alasmari A, Banu S, Sultan T, Saleh MM, Alkuraya H, Salih MA, Aldhalaan H, Ben-Omran T, Al Musafri F, Ali R, Suleiman J, Tabarki B, El-Hattab AW, Bupp C, Alfadhel M, Al Tassan N, Monies D, Arold ST, Abouelhoda M, Lashley T, Houlden H, Faqeih E, Alkuraya FS. Expanding the genetic heterogeneity of intellectual disability. Hum genetics. 2017;136(11-12):1419–29. 10.1007/s00439-017-1843-2 28940097

[76] Chen B, Chou HT, Brautigam CA, Xing W, Yang S, Henry L, Doolittle LK, Walz T, Rosen MK. Rac1 GTPase activates the WAVE regulatory complex through two distinct binding sites. eLife. 2017;6 10.7554/eLife.29795. 10.7554/eLife.29795 PMC5614565 28949297

[77] Litschko C, Linkner J, Bruhmann S, Stradal TEB, Reinl T, Jansch L, Rottner K, Faix J (2017). Differential functions of WAVE regulatory complex subunits in the regulation of actin-driven processes. Eur J Cell Biol.

[78] Zhu S, Dai J, Liu H, Cong X, Chen Y, Wu Y, Hu H, Heng BC, Ouyang HW, Zhou Y (2015). Down-regulation of Rac GTPase-activating protein OCRL1 causes aberrant activation of Rac1 in osteoarthritis development. Arthritis Rheumatol.

[79] van Rahden VA, Brand K, Najm J, Heeren J, Pfeffer SR, Braulke T, Kutsche K (2012). The 5-phosphatase OCRL mediates retrograde transport of the mannose 6-phosphate receptor by regulating a Rac1-cofilin signalling module. Hum Mol Genet.

[80] Tejada-Simon MV (2015). Modulation of actin dynamics by Rac1 to target cognitive function. J Neurochem.

[81] Huang GH, Sun ZL, Li HJ, Feng DF (2017). Rho GTPase-activating proteins: regulators of rho GTPase activity in neuronal development and CNS diseases. Mol Cell Neurosci.

[82] Bongmba OY, Martinez LA, Elhardt ME, Butler K, Tejada-Simon MV (2011). Modulation of dendritic spines and synaptic function by Rac1: a possible link to fragile X syndrome pathology. Brain Res.

[83] Katrancha SM, Wu Y, Zhu M, Eipper BA, Koleske AJ, Mains RE. Neurodevelopmental disease-associated de novo mutations and rare sequence variants affect TRIO GDP/GTP exchange factor activity. Hum Mol Genet. 2017;26(23):4728–40. 10.1093/hmg/ddx355 PMC5886096 28973398

[84] Lelieveld SH, Reijnders MR, Pfundt R, Yntema HG, Kamsteeg EJ, de Vries P, de Vries BB, Willemsen MH, Kleefstra T, Lohner K, Vreeburg M, Stevens SJ, van der Burgt I, Bongers EM, Stegmann AP, Rump P, Rinne T, Nelen MR, Veltman JA, Vissers LE, Brunner HG, Gilissen C (2016). Meta-analysis of 2,104 trios provides support for 10 new genes for intellectual disability. Nat Neurosci.

[85] Datta D, Arion D, Roman KM, Volk DW, Lewis DA (2017). Altered expression of ARP2/3 complex signaling pathway genes in prefrontal layer 3 pyramidal cells in schizophrenia. Am J Psychiatry.

[86] Redlich S, Ribes S, Schutze S, Eiffert H, Nau R (2013). Toll-like receptor stimulation increases phagocytosis of *Cryptococcus neoformans* by microglial cells. J Neuroinflammation.

[87] Guerra CR, Seabra SH, de Souza W, Rozental S (2014). *Cryptococcus neoformans* is internalized by receptor-mediated or 'triggered' phagocytosis, dependent on actin recruitment. PloS one.

[88] Johnston SA, May RC (2013). Cryptococcus interactions with macrophages: evasion and manipulation of the phagosome by a fungal pathogen. Cell Microbiol.

