# Supplementary figures and images for: Modeling the neuropsychiatric manifestations of Lowe syndrome using induced pluripotent stem cells: defective F-actin polymerization and WAVE-1 expression in neuronal cells
Source: Mol Autism. 2018 Aug 15;9:44. doi: 10.1186/s13229-018-0227-3 (PMC6094927; doi:10.1186/s13229-018-0227-3)

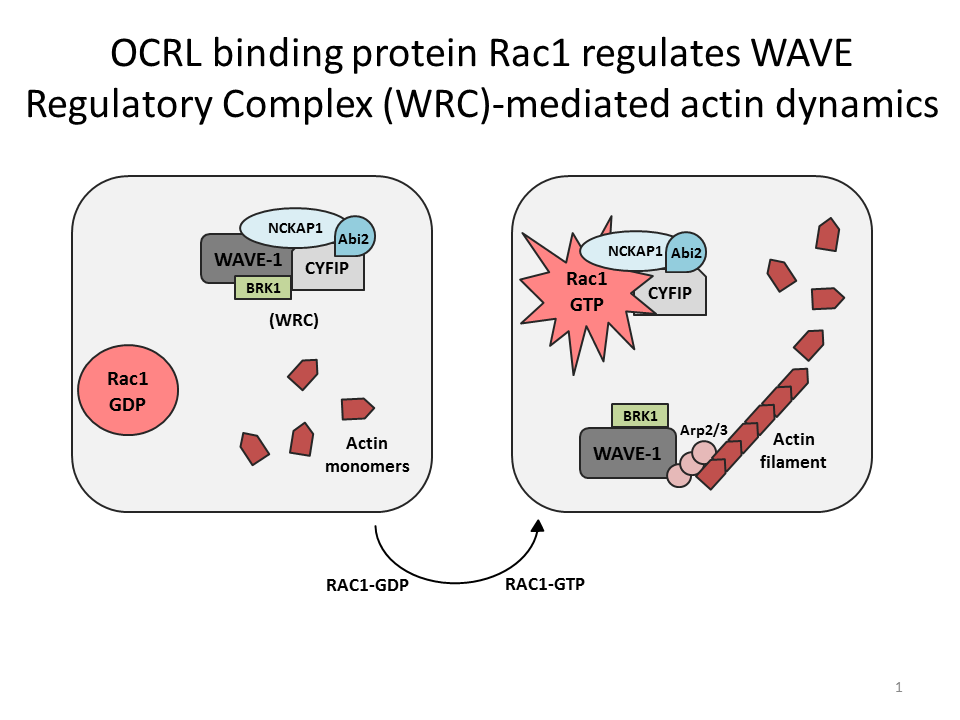

Supplement: Supplementary file 1 — Figure S1. WAVE-1 regulatory complex. Adapted from ref. 89. (TIF 89 kb) [file 13229_2018_227_MOESM1_ESM.tif]
